# Born Too Soon: Integration of intersectoral interventions for impact on preterm birth

**DOI:** 10.1186/s12978-025-02043-9

**Published:** 2025-06-23

**Authors:** Etienne V. Langlois, Maria El Bizri, Kelly Thompson, Amy Reid, Merette Khalil, Giulia Gasparri, Joy E. Lawn, Teesta Dey, Judith Robb-McCord, Yousra-Imane Benaskeur, Ana Bonell, Amanuel Gidebo, Elaine Scudder, Sophie Marie Kostelecky, Patricia Machawira, Lars Gronseth, Rajnish Prasad, Diplav Sapkota, Priya Soma Pillay, Bina Valsangkar, Bo Jacobsson, Marleen Temmerman

**Affiliations:** 1 Partnership for Maternal, Newborn & Child Health (PMNCH), World Health Organization (WHO), Geneva, Switzerland; 2 YourEgyptianDoula, Cairo, Egypt; 3 London School of Hygiene & Tropical Medicine, London, UK; 4 Sabin Vaccine Institute, Washington D.C., USA; 5 Children’s Hospital of Eastern Ontario, Ottawa, Canada; 6 Medical Research Council Unit The Gambia at LSHTM, Fajara, The Gambia; 7 World Vision, Mississauga, ON Canada; 8 International Rescue Committee, New York, USA; 9 United Nations Educational, Scientific and Cultural Organization, Johannesburg, South Africa; 10 The Norwegian Agency for Development Cooperation, Oslo, Norway; 11 United Nations Entity for Gender Equality and the Empowerment of Women, Bangkok, Thailand; 12 Scaling Up Nutrition, Geneva, Switzerland; 13 University of Pretoria, Pretoria, South Africa; 14 JHPIEGO - Johns Hopkins Program for International Education in Gynecology and Obstetrics, Maryland, USA; 15 The International Federation of Gynecology and Obstetrics, Gothenburg, Sweden; 16 Aga Khan University, Nairobi, Kenya

**Keywords:** Intersectoral interventions, Maternal health, Newborn health, Preterm birth, Equity

## Abstract

**Progress:**

The last two decades have seen a growing focus on intersectoral interventions to improve maternal and newborn health and well-being outcomes, as reflected in efforts to achieve the Millennium Development Goals (MDGs) and advance the Sustainable Development Goals (SDGs). Preterm births are linked to cross-sectoral determinants that affect health outcomes and human capital across the life-course, necessitating an intersectoral approach that addresses these multifaceted challenges.

**Programmatic priorities:**

Recognizing that social, biological and economic determinants significantly influence health outcomes, it is critical that robust health systems are reinforced by a comprehensive intersectoral approach. Evidence suggests that the factors influencing preterm birth, and the health of small and sick newborns are vast and varied, requiring interventions that address equity and rights, education, economic factors, environmental conditions, and emergency responses, i.e., a new framework entitled "five Es".

**Pivots:**

Improving outcomes for newborns, including preterm and small for gestational age babies, and preventing stillbirths, requires enhanced measurement and accountability within intersectoral programs across the 'five Es'. Investment in equity-focused, gender-transformative, and rights-based policies and programs across various sectors is crucial. Priority areas include ensuring equitable and inclusive education, particularly comprehensive sexual and reproductive health education; developing innovative financing schemes that protect and support families with complicated pregnancies and vulnerable infants; creating environmentally adaptive systems that prioritize maternal and newborn health; and implementing emergency response plans that guarantee the continuity of maternal and newborn health services. Evidence-based intersectoral interventions offer a promising pathway to reducing preterm births and improving health outcomes across generations. By addressing the five Es, intersectoral interventions can create a healthier future for preterm babies, children, adolescents, women, and society as a whole.

**Supplementary Information:**

The online version contains supplementary material available at 10.1186/s12978-025-02043-9.

## Plain Language Summary

Improving health requires action from many different areas beyond the health sector. Factors like income, environment, education, and access to quality services all impact the health of women and babies. This paper specifically looks at how these factors affect preterm births—babies born before 37 weeks—and how working across sectors can help address this challenge.

Many factors influence preterm births and newborn health, so solutions must focus on equity and rights, education, the environment, economy, and emergency response—the "five Es." To improve survival rates and quality of life for preterm babies, we need better tracking, coordination, and accountability for programs addressing these areas. Investing in these efforts leads to better outcomes and prevents preterm births.

To support vulnerable newborns, key priorities include making education more inclusive, especially sexuality education; supporting families with preterm babies through protective financial systems; improving health system capacity to adapt to environmental challenges; and creating emergency response plans that prioritize mothers and newborns. By focusing on the five Es, intersectoral approaches can reduce preterm births and improve health for future generations.

**Supplementary Information:**

The online version contains supplementary material available at 10.1186/s12978-025-02043-9.

## Key findings

**Progress:**

• In the past two decades, there has been a growing focus on intersectoral interventions to improve maternal and newborn health, to firstly achieve the MDGs and advance the SDGs.

• Intersectoral action can reduce preterm births, benefiting mothers, babies, and future generations while enhancing human capital.

**Programmatic priorities:**

• Intersectoral determinants affect women and their vulnerable newborns throughout the life-cycle.

• The "five Es"—equity and rights, education, economy, environment (including nutrition and climate) and emergencies—need urgent attention through targeted evidence-based interventions.

**Pivots:**

It is essential to invest in building equity-focused, gender-transformative and rights-based policies and programmes across sectors, prioritizing:

• equitable and inclusive education, including comprehensive sexuality education;

• innovative financing schemes that protect and support families with preterm babies;

• environmentally adaptive systems that prioritize maternal and newborn health;

• emergency preparedness and response plans that ensure the continuation of maternal and newborn health services

**Supplementary Information:**

The online version contains supplementary material available at 10.1186/s12978-025-02043-9.

## Introduction

This paper is part of the Born Too Soon supplement and focuses on intersectoral interventions to improve preterm birth and the health of mothers and newborns, and aims to highlight the need for an intersectoral approach that addresses the multifaceted challenges of preterm birth by focusing on equity and rights, education, economy, environment (including nutrition and climate) and emergencies ("the five Es").

The papers in this supplement were developed from the report “*Born Too Soon: A decade of action on preterm birth”* [1]. The report was part of a campaign to create a movement for preterm birth, linked to the need to accelerate progress for maternal and newborn health and stillbirths, noting slowing of momentum, with flatlining progress for preterm birth being foundational. Content derives from evidence synthesis of new data, literature reviews and case studies highlighting policy, implementation and community perspectives, collated into three themes: (1) *progress* particularly in the last decade; (2) programmatic priorities based on evidence; and (3) pivots needed to accelerate change in the decade ahead. The first paper in this series summarises the definitions and terminology [2].

## Main body

### Progress

**Fig. 1:**
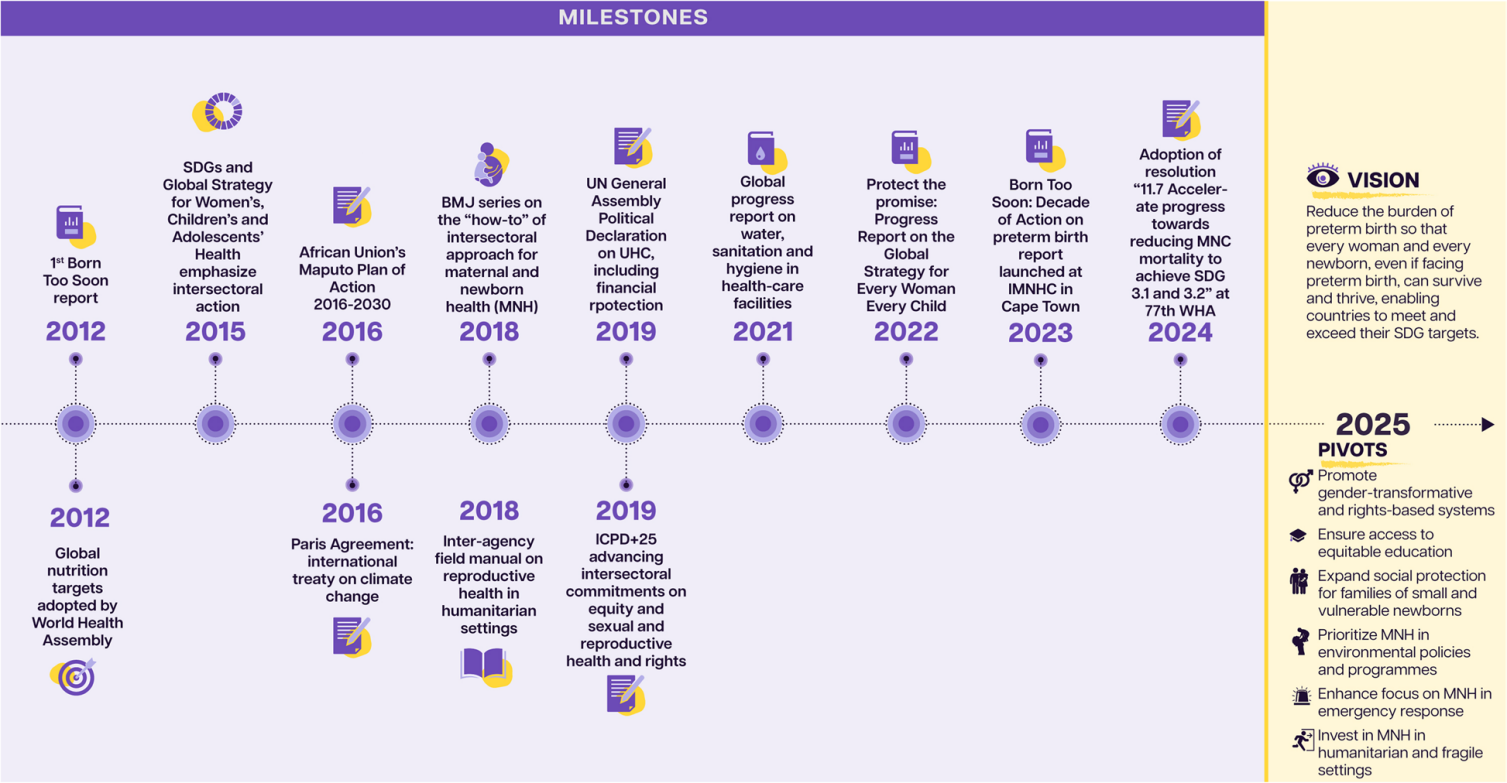
Intersectoral action on preterm birth: progress over the past decade and vision for the future

Remarkable progress in maternal and child survival over recent decades wasn't solely due to health sector developments. Between 1990 and 2010, the 50% reduction in under-5 mortality came from investments in economic growth and education [3, 4], highlighting the importance of intersectoral approaches as featured in the 2030 Agenda for Sustainable Development and the Global Strategy for Women’s, Children’s and Adolescents’ Health (2016–2030). Figure 1 presents policy and implementation milestones relating to intersectoral action in the past decade.

Amid the emerging polycrisis of climate change, conflicts, rising living costs, and pandemic risks, intersectoral action is widely recognized as essential to achieving health-related goals, including those concerning preterm birth and the survival of small and sick newborns [5]. The Sustainable Development Goals (SDGs) have one health goal (SDG3) linked to 16 intersectoral goals and shocks from the polycrisis threaten to reverse progress on health-enhancing SDGs, including those related to education and gender equality [6].

The factors influencing preterm birth and overall newborn health are vast and varied and the understanding of how diverse determinants impact maternal and newborn health outcomes, particularly in preterm babies, is still evolving. For example, the Nurturing Care Framework is a cross-sectional framework that provides strategic guidance for the holistic development of children from pregnancy to age 3 encouraging sectors such as health, nutrition, education, labour, finance, water and sanitation, and social and child protection to collaborate in new ways to address the needs of young children [7].

### Programmatic priorities

**Fig. 2:**
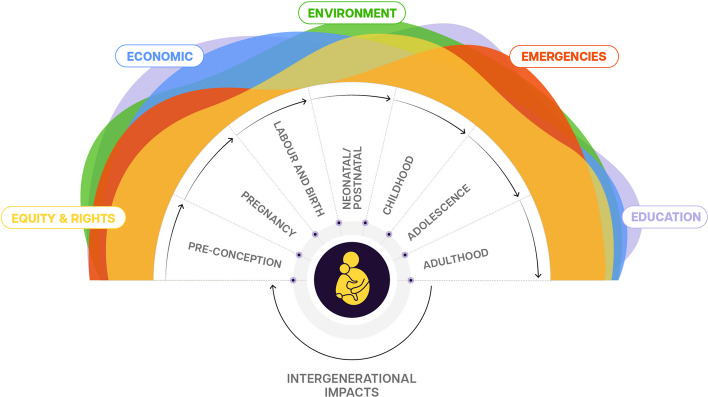
The “5 Es” of intersectoral influence on preterm birth

This paper presents a novel framework to support a coordinated and comprehensive intersectoral approach to address preterm birth and showcases the integration and co-production of interventions across sectors [8]. The new framework, introduced in the 2023 Born Too Soon report, outlines five factors most profoundly affecting women at risk of preterm birth: equity and rights, education, economy, environment, and emergencies (the"five Es") (Fig. 2).

Preventing preterm birth and stillbirth, while improving newborn outcomes, requires dedicated efforts and resources to implement evidence-based interventions in an integrated manner that fosters effective collaboration across all sectors, outlined in Table 1. Paper 7 [9] further underscores the significance of intersectoral interventions as powerful'health enhancers'and highlights the importance of prioritizing cross-sector collaboration in policy and implementation to improve outcomes for mothers and babies.

**Table 1:** Components of an intersectoral approach to address preterm birth across the life-course

5 Es	Risk factors for preterm birth	Interventions (general)	Interventions (with evidence specific to preterm birth)
Equity and rights	Gender-based violence Child marriage Female genital mutilation (FGM)	Laws, policies and programmes to address harmful gender practices such as early and forced marriage, FGM and gender-based violence [10] Gender-transformative laws, policies and programmes that advance gender equality and women’s bodily autonomy and agency [11] Policies and laws that support increased women’s representation and leadership at all levels of government and decision-making [12]	Prevent child marriage [13] and FGM [14] Reduce intimate partner violence, including by promoting positive masculinity[15]
Education	Reduced educational attainment, especially of girls and women	Laws, policies and programmes that support the advancement of girls’ education and counter harmful gender and social norms [16] Transformative comprehensive sexuality education programmes that promote gender equality and prevent early and unintended pregnancy [17, 18]	Social protection to enable girls to remain in school beyond primary education [19] Implementation of nurturing care to support neurological development, especially for at-risk newborns [7]
Economic	Out-of-pocket payments, poor access to high-quality care, lack of financial or social protection	Cash transfer programmes that cover nutrition, housing, education and access to high-quality health care[20, 21] Policies that promote sustainable and equity enhancing financial incentives [22]	Parental leave and entitlements that address the special needs of mothers, fathers and other primary caregivers of preterm or low birth-weight babies [23] User-fee alleviation and subsidization of essential care [24] Universal child health benefit, e.g., cash transfers for new mothers and families [25] Investments in systems-level interventions addressing the underlying causes of preterm birth and low-birth-weight babies, including gender empowerment and education [17, 18], programmes addressing adolescent SRHR, and prevention of child marriage and teenage pregnancy [26] Increased resources for better health system performance and targeted social protection measures to improve financial risk protection for families of preterm and low birthweight babies [19]
Environment	Climate threats, lack of WASH, and unmet nutritional needs, including suboptimal breastfeeding	Climate-resilient and environmentally sustainable health systems that support uninterrupted delivery of maternal, newborn and child health-care services [27] Laws and regulations to protect communities from exposure to pollutants known to be harmful to human health, including reproductive health [28] Improved implementation of existing WASH strategies and frameworks, such as the WHO strategy on WASH and the Every Newborn Action Plan [29] Implementation of gender-responsive national nutrition plans [30]	Resources and services for pregnant and postnatal women living in areas affected by climate change [31] Integration of maternal and perinatal care into climate mitigation and adaptation plans [32] Implementation of nutritional guidance, including exclusive breastfeeding[33] Promoting positive masculinity (sharing responsibility of household chores) to reduce exposure to air pollutants[34]
Emergencies	Humanitarian and natural disasters, conflicts	Increased financial and human resources to implement best practices for integrating maternal and newborn health services in these settings, as well as capacity building and infrastructure investments [35] Implementation of gender-responsive emergency response plans that ensure maternal and newborn health services[36]	Implementing existing guidance on delivering life-saving maternal and newborn care during responses[37–40]; maintaining routine maternal and newborn health services during disease outbreaks[41]; supporting self-care interventions; and capturing core indicators relating to preterm birth and low-birth-weight babies Expansion of women’s access to social protection programmes, especially in emergencies where women struggle to obtain adequate nutrition[41]

#### Equity and rights

Factors affecting women’s equity and rights, including the lack of autonomy, agency, and economic independence, are often compounded by intersecting factors such as racism and gender discrimination. These systemic inequalities contribute to the marginalization of women, impairing their health, their children’s health and overall societal progress. For example, racial discrimination is associated with higher rates of maternal mortality, preterm birth and low birth weight [42].

Gender-based violence (GBV) is also associated with higher rates of preterm birth [43–48]. Teenage mothers are at particular risk, as their gender and age make them twice as vulnerable to GBV, which stems from intersectional systems of oppression, exclusion and discrimination [49]. The consequences of child marriage, including lack of education and unemployment, are lifelong, and propagate an intergenerational cycle of poorer health outcomes [50]. A study in Bangladesh showed that girls married under 18 years of age were 3.18 times more likely to give birth preterm [51]. Additionally, studies show that women who have had female genital mutilations are more likely to give birth prematurely [52] and lead to poor neonatal outcomes [53].

Policies and laws that address equity and rights in maternal and newborn health outcomes are essential and discussed further in Paper 3 of this supplement [54]. Key measures include laws against early and forced marriage [55], female genital mutilation (FGM), and GBV, which directly impact the health and safety of women and girls [10].

Gender-transformative policies also promote bodily autonomy by supporting access to reproductive health services like family planning and safe abortion [11]. Additionally, policies that increase women’s representation in leadership roles and in decision-making [12] and promoting positive masculinity [15], create safer and more equitable environments for mothers and children. Programs that address one point of inequality can also reduce others.

#### Education

**Fig. 3:**
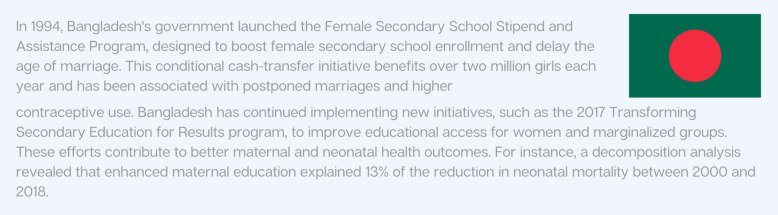
Country Snapshot: Bangladesh’s Incentives for Women’s Education Drive Positive Impacts on Maternal and Neonatal Mortality [61]

Education is a cornerstone for increasing socioeconomic status and, in turn, a key predictor of the health of both women and their babies. Reduced educational attainment, or high school completion, has consistently been shown to be associated with a 10–57% increase in preterm birth [56–59]. This is especially true in the case of adolescent pregnancies, where rates are already driven and compounded by lack of economic opportunities, pervasive inequity and malnutrition. For adolescents, lower levels of partner’s education are also associated with adverse health outcomes for both mother and baby, as well as worse birthing experiences [60]. Figure 3 details how Bangladesh incentivized women’s education, especially in rural areas, which led to downstream positive effects on neonatal and maternal mortality.

**Fig. 4:**
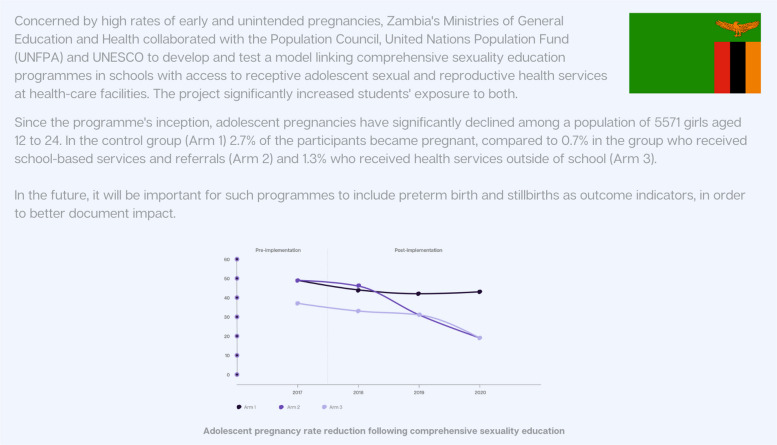
Country snapshot: Reducing adolescent pregnancy through comprehensive sexuality education in Zambia [17]

Policies and laws aimed at improving education are essential for directly enhancing maternal and newborn health outcomes. This includes initiatives that advance girls’ education and counter harmful gender and social norms [16]. Increased access to education, particularly secondary education including comprehensive sexuality education for adolescent girls, along with social protection measures [19], has been shown to promote gender equality [62], reduce early and forced child marriage and reduce adolescent pregnancy. Figure 4 highlights the approach taken in Zambia to reduce adolescent pregnancy.

#### Economic

The interrelationship between poverty and health, and the impact of poor health on economic development, are well established [63]. More than 90% of extremely preterm babies (less than 28 weeks) born in low-income countries die within the first few days of life, in comparison to less than 10% of extremely preterm babies that die in high-income settings [64]. Stark disparities in neonatal and stillbirth outcomes also occur across income levels within countries [65].

**Fig. 5:**
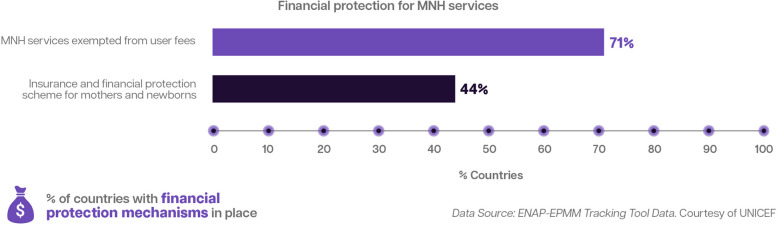
Financial protection for maternal and newborn health services, 2022 [67]

Out-of-pocket payments (OOPs), which are common in countries of all income levels, can put the greatest pressure on the poorest, and catastrophic health spending can push vulnerable families into poverty [66]. For families of preterm and other sick newborns, OOPs may be required for a baby’s hospital stay and treatment, as well as indirect costs such as travel and accommodation for family members. OOPs tend to have the greatest impact on poor and marginalized groups. Of the 106 countries surveyed in 2022 using the ENAP-EPMM Tracking Tool, 59 do not have an insurance scheme that covers all pregnant women and mothers (24% are low-income countries, 44% are lower-middle-income countries, 30% are upper-middle-income countries, and 2% are high-income countries) (Fig. 5) [67].

**Fig. 6:**
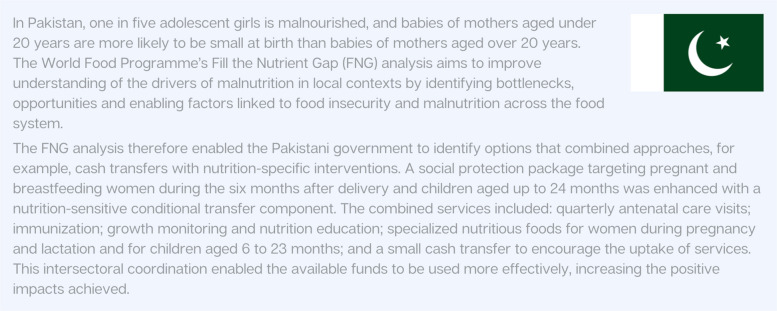
Country Snapshot: Social Protection Programs as Interventions to Meet Nutritional Needs of Mothers in Pakistan [68]

Social protection measures are also vital for the families of preterm babies. The 2022 World Health Organization (WHO) preterm birth recommendations cited ‘family involvement’ as key to improving routine preterm care, which should start in facilities and continue post-discharge [23]. However, some families cannot fulfil these essential roles without social protection. As exemplified in Paper 3 of the Supplement [54], parental leave and entitlements are necessary to address the special needs of mothers, fathers, and other primary caregivers of preterm babies [23]. Figure 6 highlights how social protection programming addressed the nutritional needs of pregnant and breastfeeding mothers and newborns in Pakistan.

Small newborns face lifelong health risks, with intergenerational effects leading to significant societal costs in human capital, productivity, and healthcare [69]. In South Asia, scaling up an evidence-based package of interventions to save the lives of newborns has been calculated to return US$ 2–17 for every US$ 1 invested [70]. In the United Republic of Tanzania, an investment case to scale up small and sick newborn care shows a potential return of US$7 in 2025 and US$9 in 2030 for every US$1 invested [71].

Evidence-based interventions, such as alleviating user fees, subsidizing essential care [24], and implementing universal child health benefits—like cash transfer programs [25]—have proven to be effective. Additional file 1 explains how Senegal removed financial barriers to delivery care, helping to increase utilization of healthcare services and ultimately lower maternal and neonatal mortality. Similarly, Additional file 2 explains how Nepal removed user fees from delivery care services and provided cash incentives to women to access maternal and neonatal health services. Countries must invest in financial protection and Universal Health Coverage (UHC) schemes and extend coverage to the most vulnerable communities, to ensure access to health services according to need, rather than ability to pay. Further investment in system-level interventions, alongside policies that promote sustainable and equity-enhancing financial incentives [22], are summarized in Table 1.

#### Environment

Emerging evidence highlights the significant influence of environmental factors at the global, national, and individual levels—such as climate change, air pollution, WASH (water, sanitation, and hygiene), and nutrition—on preterm birth and linked perinatal outcomes, such as stillbirth [72]. The intersections of climate change with food systems, nutrition, and migration also have important impacts on health outcomes [73].

##### Climate change and air pollution

Climate change has harmful impacts during the perinatal period [72]. It increases the risk of preterm birth by direct pathways, such as air pollution caused by burning fossil fuels; extreme heat exposure [74] and extreme weather events, such as drought, often intersecting with displacement and conflict [75]. Growing evidence suggests that high temperatures increase risks of preterm birth and stillbirth. A 2024 study shows that for each 1 °C increase in temperatures, the odds of preterm birth have an average increase of 5% [75].

Recent estimates suggest that household air pollution is an attributable factor for 15.6% of all low-birth-weight babies and 35.7% of all preterm births, notably in low-income countries [75]. Countries most affected by climate change have in fact contributed the least to the crisis [76] with 91% of deaths of preterm babies related to air pollution occurring in low- and middle-income countries (LMICs), despite high- income countries contributing more to climate change [77].

**Fig. 7:**
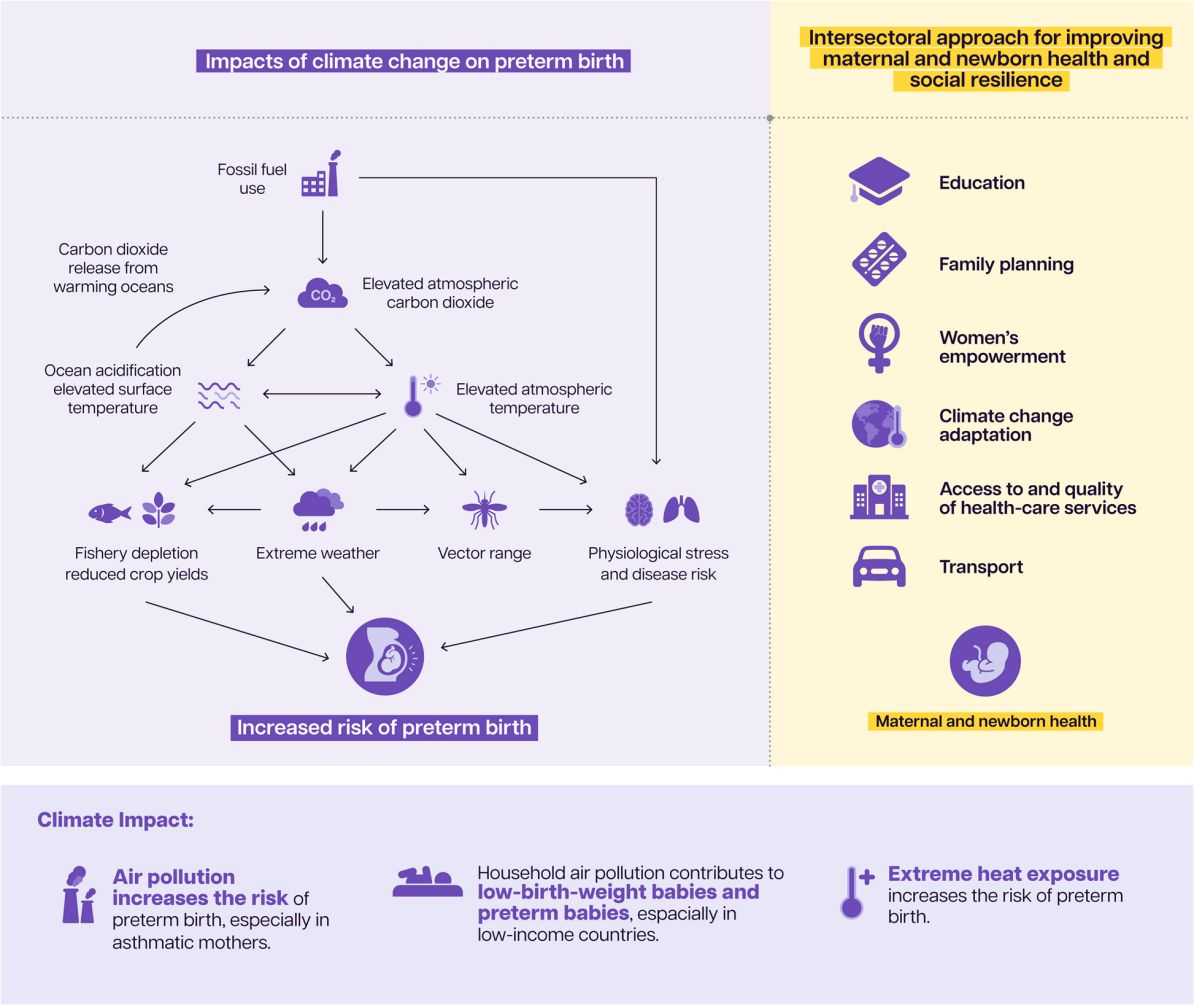
Impacts of climate change on maternal and newborn health

Figure 7 provides an overview of the impact of climate change on women and newborns, and key areas where intervention is needed to reduce that impact.

There is an urgent need to invest in climate-resilient and environmentally sustainable health systems that can ensure uninterrupted maternal, newborn, and child health care services, even in the face of climate change [27]. Strengthening laws and regulations to protect communities from harmful pollutants is equally important, particularly when considering their impact on reproductive health [28]. As outlined in Table 1, countries may consider integrating maternal and perinatal care into broader climate mitigation and adaptation strategies [32].

##### Nutrition

The availability and quality of food in each environmental context shape nutritional intake, with factors like food insecurity, poverty, and agricultural practices influencing maternal and neonatal health, thereby affecting growth, immunity, and development. There is a significant correlation between the nutritional status of pregnant women and birth weight of the baby [78]. Conversely, maternal obesity is associated with an increased risk of preterm birth [79].

Nutritional deficiencies, particularly iron deficiency anaemia, can lead to preterm delivery and low birth weight [30], as well as decreased iron in the baby which may lead to impaired child development [80]. Globally, women and girls comprise the majority (60%) of people with chronic malnutrition, and nearly 30% of women of reproductive age (15–49 years) suffer from iron deficiency anaemia [81]. In the 12 countries hardest hit by the current food and nutrition crisis, the number of acutely malnourished pregnant and breastfeeding adolescent girls and women increased by 25% between 2020 and 2022 [82].

Preterm birth is one of two underlying causes of low birth weight (small-for-gestational age being the other) [83]. Low-birth-weight newborns are at a higher risk of becoming children who experience stunting, wasting, and developmental delays. They are also more likely to become undernourished adolescents and, eventually, undernourished adults – perpetuating a vicious cycle [33]. Low-birth-weight newborns are also more likely to have adult-onset chronic diseases, such as hypertension and diabetes [84].

Recent evidence has shown the benefits of cash transfers on positive childhood nutritional outcomes, bolstering support for delivering child nutrition and social protection programs together [85, 86]. While pending further research, early evidence shows that nutritional supplement interventions can have promising benefits for reducing newborn mortality and morbidity outcomes [87].

**Fig. 8:**
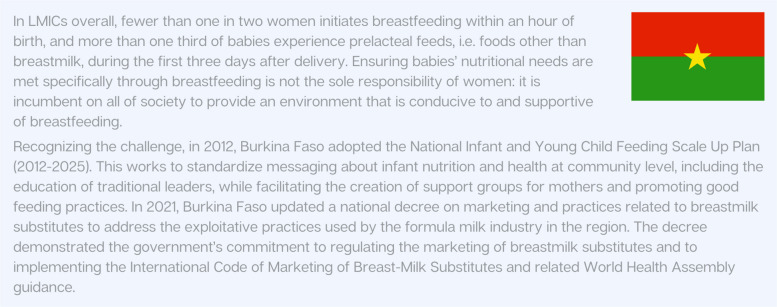
Country snapshot: Intersectoral approach to babies’ nutrition and support breastfeeding in Burkina Faso [89]

Breastfeeding is a high-impact practice for all newborns and especially important for those who are preterm, with extra support needed to enable exclusive and optimal feeding for six months. Figure 8 presents an example of intersectoral support for breastfeeding and good practices to mitigate the impact of marketing breast milk substitutes in Burkina Faso. Intersectoral action is crucial, including parental leave and greater policy momentum to address social determinants and inappropriate marketing practices [88]. The implementation of gender-responsive national nutrition plans [81] and nutritional guidance, including exclusive breastfeeding, are essential interventions to improve maternal and newborn outcomes through proper nutrition.

##### Water, sanitation and hygiene (WASH)

The state of water, sanitation, and hygiene in a household or community determines exposure to pathogens and infectious diseases, creating an environment that can either support maternal and newborn health or contribute to negative health outcomes. Lack of access to clean drinking water and poor sanitation negatively affect the health of women and babies. For example, exposure to *Listeria monocytogenes* bacteria in water, for which the infection rate is more than 18 times higher in pregnant women, is associated with miscarriage and preterm birth [90].

**Fig. 9:**
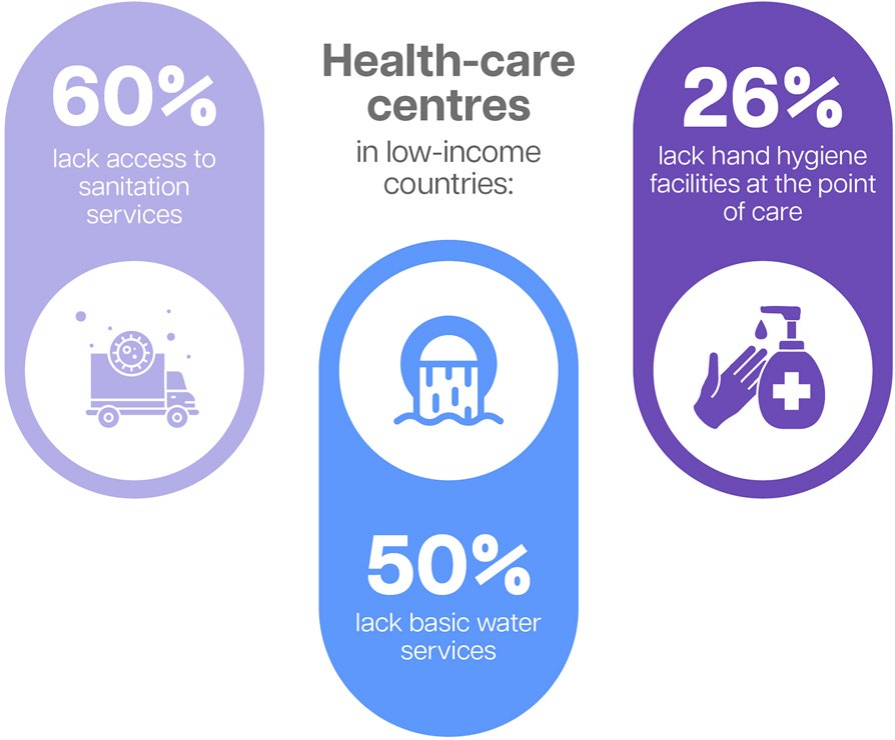
WASH in health-care centres in low-income countries [93]

Lack of sanitation facilities also affects women and newborns negatively, yet almost half the world’s population does not have access to safely managed sanitation [91]. For example, a study in India found that women who practise open defecation, as well as those without a place to wash their hands, are likely to experience poorer pregnancy outcomes than those with such access [92]. Infections acquired in health-care facilities also present a serious risk to women and newborns. Figure 9 shows the three top gaps in WASH services in health-care facilities in low-income countries.

**Fig. 10:**
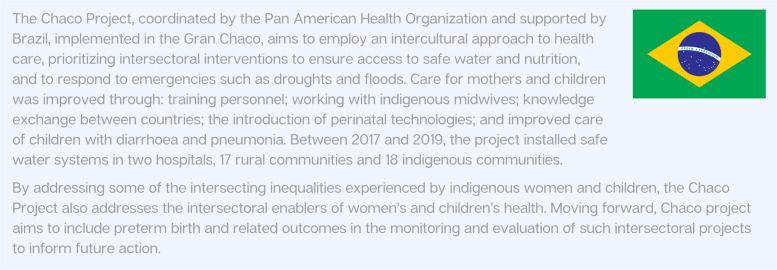
Country snapshot: Ensuring Safe Water and Nutrition for Mothers and Children in Gran Chaco [96–98]

Addressing the impact of WASH on maternal and newborn health requires culturally sensitive programs that improve women's access to clean and safe water and toilet facilities, as highlighted in Fig. 10. Interventions at the health-care facility level may include installing water systems to ensure running water in maternity wards, as well as building post-delivery washrooms. At the community level, interventions may include training for community artisans to construct low-cost improved latrines, and door-to-door health education on sanitation and hygiene practices [29]. Strengthening the implementation of existing WASH strategies, such as the WHO strategy on WASH [94] and the Every Newborn Action Plan [95], is also key to ensuring sustainable improvements.

#### Emergencies

Emergencies including conflicts, pandemics and epidemics may have devastating consequences for the health of women and newborns, including preterm babies. For instance, 25 countries that have a 2024 UN Humanitarian Appeal account for 58% of global maternal deaths, 38% of newborn deaths, and 36% of stillbirths [99]. Moreover, while progress to reduce maternal deaths is stagnating worldwide, countries responding to humanitarian crises are lagging furthest behind [100]. Mothers exposed to armed conflict have an increased risk of giving birth to low birthweight babies [37].

**Fig. 11:**
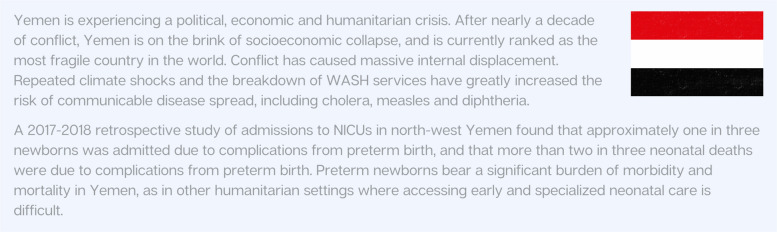
Country snapshot: Impact of emergencies on preterm birth and small and sick newborns in Yemen [106]

In addition, risk factors for poor maternal and neonatal outcomes are likely to increase substantially in humanitarian settings: examples include exposure to acute and chronic stressful events [101, 102], gender-based violence [103, 104], infectious diseases and unsanitary conditions [105], disrupted access to care [38] and displacement [101]. Figure 11 highlights the impact of emergencies on preterm births in Yemen.

Capacity to deliver the specialized and highly technical care needed for small and/or sick newborns is often limited in humanitarian-affected settings and within responses where health systems are already fragile and under-resourced. Among respondents to a 2018 survey of Global Health Cluster partner agencies [107], fewer than half (47%) reported having the technical capacity to provide essential newborn care during an emergency response, including care of low-birthweight and preterm babies.

As exemplified in Papers 4 and 5 of this Supplement [108, 109], access to high-quality maternal and newborn health services in *all* settings is key to improving outcomes like preterm birth prevention and achieving Universal Health Coverage. In humanitarian settings, increased financial and human resources, capacity building, and infrastructure are needed to integrate these services effectively[38]. Gender-responsive emergency plans should ensure continuity of care, while existing guidelines [41] for life-saving maternal and newborn care must be followed, even during disease outbreaks [110]. Other evidence-based interventions are listed in Table 1.

### PIVOTS

Siloed, single-sector approaches, limited financing and poor data on preterm births and stillbirths have historically resulted in poorer outcomes for mothers, newborns and families. The following pivots are vital to prevent preterm birth, protect small and/or sick newborns and their families, and ensure greater accountability.

#### Pivot 1: equity through gender-transformative and rights-based policies and programmes across sectors

Government officials and community leaders should address harmful gender and social norms and ensure that programmes counter historic inequities, upholding the rights of the most marginalized. Policies and frameworks should promote sexual and reproductive health and rights and women’s bodily autonomy and agency, specifically addressing harmful gender and social norms, such as child marriage, intimate partner violence and sexual violence, and female genital mutilation.

#### Pivot 2: education that is inclusive through the life-course

A life-course approach is essential to ensure a healthy start, support early childhood development, and retain adolescent girls in secondary education. This includes integrating the principles of the Nurturing Care Framework to ensure that early childhood development interventions, such as responsive caregiving, good health, adequate nutrition, and opportunities for early learning, are prioritized during the foundational early years. Promoting equitable access to inclusive, high-quality education requires addressing gender disparities in educational attainment, providing comprehensive sexuality education to transform gender and social norms, and creating safe, harassment-free school environments with adequate privacy for menstruating girls.

#### Pivot 3: economic investments that prioritize co-financing across sectors

Greater investment in financing for preventing preterm birth and caring for small and/or sick newborns, through equity-focused and cross-sectoral financing models, is essential for paving the way towards comprehensive care coverage for mothers and babies. This includes ensuring that maternal and newborn care are included in universal health coverage and insurance schemes, avoiding or minimizing out-of-pocket payments, and providing social protection and extra support for families of small and/or sick newborns. Pooled budgets across sectors will ensure that available funding has a synergistic impact by optimizing public spending and directing funding to health-enhancing programs. Additionally, interventions that prevent preterm births need to be embedded in health financing reforms and strategies.

#### Pivot 4: environmental awareness and action

It is essential to use an intersectional lens when considering the populations who are most vulnerable to environmental conditions. Bearing the greatest burden and highest risks, the specific needs and vulnerabilities of women, children and newborns must be considered when building and strengthening systems to provide nutrition, WASH, clean air and climate adaptation responses.

Improving access to safe water and sanitation, ensuring clean air, ending hunger, and addressing malnutrition across the life-course are essential. Furthermore, women, newborns, children and adolescents should be explicitly prioritized in climate adaptation and mitigation strategies and policies.

#### Pivot 5: emergency preparedness and response

Improve prevention, identification, and care for preterm births and small and/or sick newborns in humanitarian responses by strengthening and expanding national and international preparedness and response plans. Ensure these plans are comprehensive and include dedicated funding for life-saving health commodities and trained responders. Develop and implement standard operating procedures and good practices to promote integration and coordination among response agencies and within cluster systems, thereby breaking down silos in response efforts. Focus on health system strengthening in fragile settings, ensuring that districts and sub-national regions are included in national efforts to improve referral pathways, infrastructure, support for healthcare providers, and data systems including monitoring of essential health services.

Yet, the integration of services across sectors is not a one-size-fits-all solution. Integrating programmes entails challenges, including the need for intersectoral coordination mechanisms, shared governance, and investments in joint planning and accountability. These factors must be carefully considered when designing intersectoral interventions. Nevertheless, greater policy coherence and strategic collaboration across sectors — where feasible and contextually appropriate — can contribute to more resilient systems that better support mothers, newborns, and families. 

## Conclusion

Addressing the challenges of preterm birth necessitates a concerted effort to break down silos and foster collaboration among various sectors. The identified"five Es"—equity and rights, education, economy, environment, and emergencies—underscore the need for a whole-of-government and whole-of-society approach that recognizes the interconnectedness of these factors. By integrating interventions across sectors, a more supportive environment for mothers, newborns, and families can be created. In times of fiscal constraints, integrated interventions offer a cost-effective approach by streamlining resource use, avoiding duplication, and maximizing the impact of limited health budgets. Different intersectoral interventions and models may be more appropriate depending on the context, institutional capacity, and resource availability. Integrated interventions for preterm birth and high-quality maternal and newborn care require careful consideration of contextual appropriateness, robust coordination, shared governance, and joint investments to support implementation.

Comprehensive, intersectoral policies and financing, including co-financing schemes across sectors, have the potential to significantly improve maternal and newborn health outcomes and accelerate progress towards Sustainable Development Goals, and yield benefits across the life-course.

## Supplementary Information

Additional file 1. Removed user fees from delivery services in Senegal contributed to lower maternal and neonatal mortality [111].

Additional file 2. Removed user fees and introduced financial incentive programs in Nepal increase access to maternal and neonatal health services [112].

## Data Availability

All data is available in the paper or in supplementary files. Additional information is available at www.borntoosoonaction.org.

## Abbreviations

ENAP: Every Newborn Action Plan
EPMM: Ending Preventable Maternal Mortality
FGM: Female Genital Mutilation
Five Es: Equity and rights, Education, Economy, Environment (including nutrition and climate), and Emergencies
GBV: Gender-based violence
LMICs: Low- and Middle-Income Countries
OOP: Out-of-Pocket Payment
SDG: Sustainable Development Goal
UHC: Universal Health Coverage
WASH: Water, Sanitation, and Hygiene
WHO: World Health Organization
