# Supplementary material for: Born Too Soon: Integration of intersectoral interventions for impact on preterm birth
Source: Reprod Health. 2025 Jun 23;22(Suppl 2):111. doi: 10.1186/s12978-025-02043-9 (PMC12186354; doi:10.1186/s12978-025-02043-9)
Supplement: Supplementary file 1 — Additional file 1. Removed user fees from delivery services in Senegal contributed to lower maternal and neonatal mortality [111]. [file 12978_2025_2043_MOESM1_ESM.docx]

**Additional File 1**

**Removed user fees from delivery services in Senegal contributed to lower maternal and neonatal mortality [111]**

In 2005, Senegal passed the National Free Delivery and Caesarean policy to mitigate financial barriers to facility-based delivery care. This policy removed user fees for normal deliveries at health posts and health centers, as well as C-sections at higher-level facilities. Evidence shows that this policy increased utilization of healthcare services, referrals to higher level care, and rates of C-section delivery. The Lives Saved analysis also suggests that the number of maternal and neonatal lives saved by C-section and other facility-based interventions (e.g. uterotonics, MgSO4, and antibiotics) increased dramatically after this policy was initiated.
