# Supplementary material for: Born Too Soon: Integration of intersectoral interventions for impact on preterm birth
Source: Reprod Health. 2025 Jun 23;22(Suppl 2):111. doi: 10.1186/s12978-025-02043-9 (PMC12186354; doi:10.1186/s12978-025-02043-9)
Supplement: Supplementary file 2 — Additional file 2. Removed user fees and introduced financial incentive programs in Nepal increase access to maternal and neonatal health services [112]. [file 12978_2025_2043_MOESM2_ESM.docx]

**Additional File 2**

**Removed user fees and introduced financial incentive programs in Nepal increase access to maternal and neonatal health services [112]**

Nepal introduced a variety of financial assistance mechanisms to minimize barriers to accessing care. In 2005, the government introduced a geography-specific cash incentive to women to offset transport costs for institutional delivery. Nepal’s Safe Motherhood (Aama Surakshya) program was expanded in 2009 to remove user fees for delivery care in facilities nationally, including for C-sections. This policy introduced additional delivery-related incentives for health workers and hospitals and provided referral funds for emergency transfer. The government also introduced an incentive for women to complete four antenatal care visits. Since the introduction of these policies, Nepal has experienced substantial increases in ANC4+ coverage and institutional delivery rates.
